# Intensity‐Modulated Proton and Carbon‐Ion Radiation Therapy in the Management of Head and Neck Sarcomas

**DOI:** 10.1002/cam4.2319

**Published:** 2019-06-23

**Authors:** Jing Yang, Jing Gao, Xianxin Qiu, Jiyi Hu, Weixu Hu, Xiaodong Wu, Chenping Zhang, Tong Ji, Lin Kong, Jiade J. Lu

**Affiliations:** ^1^ Department of Radiation Oncology Shanghai Proton and Heavy Ion Center Shanghai China; ^2^ Division of Research and Development SPHIC Shanghai China; ^3^ Department of Oral & Maxillofacial‐Head & Neck Oncology, Shanghai Stomatology Key Laboratory Affiliated Ninth People's Hospital, School of Medicine Shanghai China; ^4^ Department of Radiation Oncology SPHIC, Fudan University Shanghai Cancer Center Shanghai China; ^5^ Present address: Shanghai Engineering Research Center of Proton and Heavy Ion Radiation Therapy 4365 Kangxin Road Pudong Shanghai 201321 China

## Abstract

**Purpose:**

We report our experience of intensity‐modulated proton and carbon‐ion radiotherapy (IMPT/IMCT) for head and neck sarcomas (HNS).

**Methods and Materials:**

An analysis of the ongoing prospective data registry from the Shanghai Proton and Heavy Ion Center (SPHIC) for patients with HNS was conducted. The 12‐ and 24‐month rates of local recurrence‐free, overall, distant metastasis‐free, progression‐free survival (LRFS, OS, DMFS, and PFS), and acute/late toxicities were calculated. The prognostic factors for the effectiveness of the treatment were also analyzed.

**Results:**

Between 7/2014 and 5/2018, 51 consecutive patients with HNS received definitive doses of IMCT (41 cases), IMPT (two cases), or their combination (eight cases). One patient had R0 resection and another treated on the Chinese Food and Drug Administration registration trial received IMPT only. Twenty‐seven patients were treated according to various dose escalation trials or institutional protocols using IMCT or IMPT + IMCT boost. Twenty‐two patients with locoregional recurrence (10 and four patients failed surgery or surgery followed by radiotherapy, respectively) or radiation‐induced second primary sarcomas (eight patients) received salvage particle radiotherapy. With a median follow‐up time of 15.7 months, four patients with second primary sarcoma died. The 1‐ and 2‐year OS, PFS, LRFS, and DMFS rates for the entire cohort were 92.9% vs 90%, 73.6% vs 57.4%, 88.4% vs 78.9%, and 84.6% vs 76.5%, respectively. Those rates for patients without prior radiotherapy were 100% vs 100%, 82.1% vs 65.8%, 93.6% vs 85.3%, and 88.4% vs 79.5%, respectively. Multivariate analyses revealed that re‐irradiation was an independent prognostic factor for both LRFS and PFS (*P *= 0.015 and 0.037, respectively). In addition, gross tumor volume (GTV) was an independent prognostic factor for PFS (*P *= 0.048). One patient experienced Grade 3 acute toxicity (oral mucositis); another experienced Grade 4 acute event (hemorrhage) which required embolization. He lately died from hemorrhage (Grade 5) at 3.4 months after the completion of treatment. No patient experienced radiation‐induced acute/late toxicity of ≥ Grade 2 otherwise.

**Conclusion:**

With few observed acute/late toxicities, IMPT/IMCT provided effective short‐term tumor control in our patients with HNS. Further investigations, preferably in a prospective fashion, will be required to confirm the efficacy and toxicities of IMPT/IMCT in this group of patients.

## 1 INTRODUCTION

Head and neck sarcomas (HNS) arise from mesenchymal tissues and represent a rare and heterogeneous disease entity. Sarcomas account for 1% of all head and neck malignancies, and ~ 10% of all sarcomas occur within the head and neck.^1^, ^2^, ^3^, ^4^, ^5^ Complete surgical resection is the cornerstone of curative treatment for this disease, regardless of histologic subtype.^6^, ^7^ However, HNS often preclude a gross‐total resection due to their proximity to and frequent involvement of adjacent critical organs. As such, the management of HNS often requires a multidisciplinary approach, combining surgery and radiotherapy (RT), and with certain diagnoses, chemotherapy.^7^

Even with aggressive local therapy, the prognosis of HNS is worse than sarcomas that originate in other anatomical locations.^8^, ^9^ When complete resection is not feasible, photon‐based RT rarely provides adequate long‐term local control, as most sarcoma subtypes are inherently resistant to conventional RT.^10^, ^11^, ^12^, ^13^, ^14^ This reduces the likelihood of long‐term survival.^11^ Dose escalation for photon‐based RT is usually not feasible, due to the dose limitation of the adjacent critical organs at risk (OARs). Additionally, patients who develop recurrences from previously irradiated HNS or de novo sarcomas secondary to previous RT for an unrelated malignancy carry a particularly dismal prognosis, given the accumulated doses received by the OARs and emergence of radioresistant tumors.

Because of these biological and technical challenges, HNS represent a warranted clinical application for proton and carbon‐ion radiotherapy (PRT and CIRT). The physical properties of proton and carbon‐ion beam are characterized by the Bragg Peak, in which little dose is absorbed along the entry path and the dose is deposited in a finite local volume, with no exit dose. This enables precise dose distributions, which are uniquely suited for tumors within complex anatomical regions.^15^ Moreover, the proton and carbon‐ion beams have higher linear energy transfer (LET) and greater relative biological effectiveness (RBE) as compared to those of photon beam.^16^, ^17^ Recently, there was research revealing that the value of RBE of carbon‐ion was 3 in animal model of soft‐tissue sarcoma.^18^ The combination of these features should, in theory, be advantageous in overcoming both anatomic limitations and sarcoma's radioresistance, thus improving the anticipated therapeutic ratio against this challenging disease. To date, there is scant data regarding the effectiveness of particle RT especially CIRT against head and neck malignancies and even less for HNS.^19^

The Shanghai Proton and Heavy Ion Center (SPHIC) began clinical application of intensity‐modulated PRT (IMPT) and intensity‐modulated CIRT (IMCT) using pencil beam scanning (PBS) technology in May of 2015.^20^ A substantial portion of this patient population had primary and recurrent (previously irradiated) head and neck malignancies including sarcomas. In this article, we report our clinical results on the use of IMPT and/or IMCT at definitive doses for the treatment of HNS.

## 2 METHODS AND MATERIALS

### 2.1 Pretreatment evaluation

Patient evaluation before particle radiotherapy included a complete history and physical examination (H&P), complete blood count (CBC) and metabolic panel, MRI of the head and neck region (CT was allowed when MRI was contraindicated), a positron emission tomography (PET)/CT, and direct or fiberoptic endoscopy if indicated.

The staging system of the Intergroup Rhabdomyosarcoma Studies (IRS) was used to stage rhabdomyosarcoma. Since prognostic grouping for AJCC staging system is not available for the 8th edition for HNS, and the French Federation of Cancer Centers Sarcoma Group (FNCLCC) grade was not available for some patients with soft‐tissue sarcoma in this series, the 7th edition was used to stage all other patients with soft‐tissue or osteo/chondrosarcoma. Radiation‐induced sarcomas were defined by tumors of a different pathology occurred within the previous RT‐treatment target volumes after a latent period of > 5 years.^21^

Chemotherapy was used at the discretion of the medical oncologist, and was usually delivered prior to the referral and after the radiotherapy.

For newly diagnosed patients without prior radiation, neck irradiation was performed if the patient already presented neck node, or if the pathology type was with ≥ 10% probability of neck node metastasis (eg, rhabdomyosarcoma, clear cell sarcoma, synovial sarcoma). For patients with recurrent or second primary HNS with neck adenopathy, neck dissection was provided. No neck radiation was applied for re‐irradiation cases.

### 2.2 Intensity‐modulated proton or carbon‐ion radiotherapy

The planning and treatment techniques of particle radiotherapy for primary and recurrent HNS at SPHIC have been previously reported.^15^, ^22^ Briefly, all patients were immobilized in the supine position with individualized thermoplastic masks. Planning CT scans without contrast from the vertex to the inferior margin of clavicular heads were performed at 1.5‐mm slice thickness. MRI‐CT fusion was performed for all patients prior to target volume delineation. The gross tumor volume (GTV) was defined as the gross tumor discovered on clinical examination or imaging studies. We defined a clinical tumor volume (CTV) boost as the GTV with 1‐3 mm margin to deliver the prescribed dose to the tumor. For patients who received surgery and/or chemotherapy prior to radiotherapy, the pretreatment tumor bed was defined as CTV.

Old RT plans were obtained for patients previously irradiated except for the two patients who developed secondary osteosarcoma after prior RT for nasopharyngeal cancer (latent period > 10 years). The doses to the OARs were identified. Recovery from previous RT dose was set at 70%, regardless of the latent time between the two courses of radiation.^23^ Doses were measured by Gy‐equivalents (GyE) to account for the RBE differences of particle radiotherapy compared to photon. The dose constraints of the OARs are based on TD5/5 described by Emami except for optic nerve (D_20_ < 30GyE), brain stem (D_max_ < 45 GyE), spinal cord (D_max_ < 30 GyE), and temporal lobes (V_40_ < 7.66cc; V_50_ < 4.66cc), which were based on previous experience from the National Institute or Radiation Science (NIRS) of Japan.^24^ Treatment planning for IMPT and IMCT was performed using the Siemens Syngo^®^ planning system (version VC11/13).

IMPT and IMCT were delivered with PBS technology. The beam arrangement varied depending on target volume geometry, and dose limits to neighboring OARs, such as those with prior radiation exposure. Treatments typically consisted of 2‐3 beams. Individual factors such as patient positioning reproducibility and/or beam angles were chosen for optimal dosimetry. Setup accuracy was confirmed with daily orthogonal x‐ray using bony landmarks as reference. Verification CT scans were typically performed on weekly basis after the second week of the radiation for changes in anatomy.

### 2.3 Follow‐up

All patients were encouraged to adhere to our institutional standardized follow‐up protocol. The first follow‐up occurs within 4‐6 weeks after the completion of radiation, every 3 months in the first 2 years, every 6 months in the following 3 years, then annually thereafter. A complete H&P, MRI of the head and neck area are required at each follow‐up session. PET/CT and other studies are ordered based on clinical evidence of metastasis, recurrence, or other concurrent diseases.

### 2.4 Data collection and statistics

All cases treated with particle RT at SPHIC were presented and discussed in the multidisciplinary tumor clinic for their diagnoses, indications, and selection of particle radiotherapy protocol (IRB registered) prior to registration. All data for diagnosis, treatment, and follow‐ups were recorded to a prospective registry and database.

Acute adverse events were scored using the CTCAE (version 4.03) and included those occurred during or within 3 months after the initiation of particle RT. Late toxicities were scored using the Radiotherapy Oncology Group (RTOG) late radiation morbidity scoring system for toxicities observed beginning at 90 days after completion of particle radiotherapy.

The duration of survival was calculated from the date of diagnosis until the date of death or last follow‐up. The time to local or distant failure was measured from the date of the start of particle RT until documented date of failure. Freedom from failure and overall survival rates were calculated using the Kaplan‐Meier method.^25^ The Cox proportional hazard analysis method was performed to determine independent predictive factors. All analyses were performed using the SPSS statistics version 18.0 software package (Chicago, IL).

The recurrent group included relapse cases after surgery, and the re‐irradiated group included radiation‐induced second primary sarcomas. Recurrence was excluded in the univariate analysis and cox proportional hazard regression analyses for the interaction with re‐irradiation.

## 3 RESULTS

### 3.1 Study population

A total of 54 consecutive and nonselected patients with HNS were treated at SPHIC from July 2014 to May 2018. Histological diagnoses were obtained for all patients with initially diagnosed and radiation‐induced second primary sarcomas. Local recurrences were diagnosed histologically or clinically using repeated imaging studies. No patient had distant metastasis (DM) at inclusion. Three patients were excluded from this analysis: Two patients developed DM (bone and brain) during their IMPT/IMCT despite their negative PET/CT scans for initial staging; another did not complete planned IMCT after four fractions due to rupture of optic artery unrelated to his malignancy or treatment. Among the remaining 51 patients, 47 were deemed unresectable and had incomplete resection (R2) or biopsy. Twenty‐nine patients presented with newly diagnosed sarcomas, 14 patients presented locoregional recurrence after previous treatment (10 had surgery only and four had RT with or without surgery) and eight patients had RT‐induced second primary sarcoma from previous RT for nonsarcoma diagnoses.  Therefore, a total of 12 patients received prior RT.

The characteristics of the patients, their disease, and treatments are detailed in Table1.

**Table 1:** Characteristics of the patients, their disease, and treatments

Characteristic	No.	%
Sex		
Male	26	51
Female	25	49
Age (years)		
Median	36	
Range	14‐68	
Histology		
Chondrosarcoma	20	39.2
Rhabdomyosarcoma	10	19.6
Pleomorphic sarcoma	3	5.9
desmoid‐type fibromatosis	2	3.9
Spindle cell sarcoma	2	3.9
Osteosarcoma	4	7.8
Others	10	19.6
Site		
Skull base	17	33.3
Nasal cavity‐paranasal sinus	15	29.4
Nasopharynx	2	3.9
Oropharynx	3	5.9
Oral cavity	5	9.8
Major salivary gland	1	2.0
Neck	3	5.9
Orbit	5	9.8
Clinical stage		
I ~ II	27	52.9
III ~ IV+recurrent	24	47.1
Primary or recurrent		
Primary	29	56.9
Recurrent/second primary	22	43.1
Re‐radiotherapy		
Yes	12	23.5
No	39	76.5
Surgery		
R0 + R1	4	7.8
R2 + biopsy + no surgery	47	92.2
Chemotherapy		
Yes	18	35.3
No	33	64.7
PT beam types		
IMPT	2	3.9
IMCT	41	80.4
IMPT + IMCT	8	15.7

Abbreviations: IMCT, intensity‐modulated carbon‐ion radiotherapy; IMPT, intensity‐modulated proton radiotherapy; KPS, Karnofsky performance status; PT, particle radiotherapy.

### 3.2 Particle radiotherapy and adjuvant treatment

**Figure 1:**
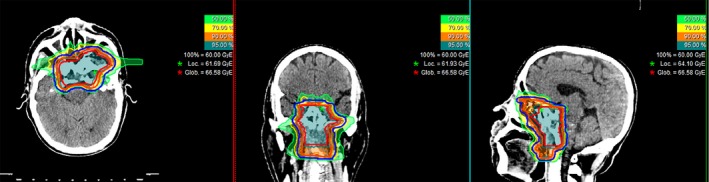
A typical IMCT treatment plan of a patient with locoregionally advanced soft‐tissue sarcoma of nasopharynx

All patients received IMPT or IMCT using PBS technology. Two patients received IMPT only: One patient was treated according to the Chinese Food and Drug Administration required registration trial which designated IMPT only, and another was treated according to our institutional post‐op protocol after complete (R0) resection for head and neck malignancies which required adjuvant IMPT only regardless of pathology. Forty‐one completed IMCT and eight completed a combination of IMPT and IMCT boost according to our dose escalation trials or standard institutional protocols. A typical treatment plan is shown in Figure 1. The details of dose/fractionation schemes were detailed in Table 2.

**Table 2:** Fractionations of IMPT/IMCT treatment scheme

Fractionation	Total dose (GyE)	Fraction (Fx)	No.
IMPT			
70GyE/35Fx	70	35	1
56GyE/28Fx	56	28	1
IMPT + IMCT			
56GyE/28Fx + 15GyE/5Fx	71	33	3
54GyE/30Fx + 18GyE/6Fx	72	36	1
50GyE/25Fx + 15GyE/5Fx	65	30	1
50GyE/25Fx + 20GyE/8Fx	70	33	2
50GyE/20Fx + 12GyE/4Fx	62	24	1
IMCT			
54GyE/18Fx	54	18	1
57.5GyE/23Fx	57.5	23	1
60GyE/20Fx	60	20	9
63GyE/21Fx	63	21	9
63GyE/18Fx	63	18	10
66GyE/22Fx	66	22	5
66.5GyE/19Fx	66.5	19	2
69GyE/23Fx	69	23	3
70GyE/20Fx	70	20	1

Chemotherapy was provided to 18 patients with rhabdomyosarcoma, undifferentiated, pleomorphic, and small round cell sarcomas, osteosarcoma and chondrosarcoma. Regimens were determined by the medical oncologists of individual patients for induction and/or concurrent chemotherapy. Only one patient with radiation‐induced undifferentiated sarcoma received concurrent chemotherapy with salvage particle RT. Six of the 12 patients who had previous RT as well as 12 of the initially diagnosed patients received chemotherapy.

### 3.3 Acute and late adverse effects

Initial and retreatment with IMCT and/or IMPT was well tolerated for all except for one patient. The most commonly observed acute adverse effects were Grade 1 or 2 mucositis and dermatitis. Only one patient experienced an acute Grade 4 event (hemorrhage). He successfully underwent embolization of the bleeding artery then completed the planned IMPT. The same patient died from hemorrhage at 3.4 months after the completion of IMCT. No other patient experienced Grade 2 or higher radiation‐induced toxicity 90 days after the initiation of particle RT except for one who had Grade 2 xerostomia due to salivary glands impairment. Profiles of acute and late toxicities were detailed in Tables 3 and 4.

**Table 3:** Types and frequency of acute toxicities

Toxicity	Grade							
	1		2		3		4	
	No.	%	No.	%	No.	%	No.	%
Mucous membrane	15	23	5	9.3%	1	1.9	0	
Skin	15	23	1	1.9%	0		0	
Hemorrhage	1	1.9	0		0		1	1.9
Tinnitus	1	1.9	0		0		0	

**Table 4:** Types and frequency of late toxicities

Toxicity	Grade									
	1		2		3		4		5	
	No.	%	No.	%	No.	%	No.	%	No.	%
Salivary glands (dry mouth)	7	13.7	1	1.9	0		0		0	
Skin	2	3.9	0		0		0		0	
Decreased hearing	4	7.8	0		0		0		0	
Decreased vision	1	1.9	0		0		0		0	
Pain	4	7.8	0		0		0		0	
Parageusia	3	5.9	0		0		0		0	
Hemorrhage	0		0		0		0		1	1.9

### 3.4 Overall survival, local and distant failure, and progression‐free survival

All patients were required to be followed‐up using our institutional follow‐up protocol. The median follow‐up time was 15.7 months (range 2.8‐56.7) for all patients. At the time of this analysis, 17 patients developed disease progression or failure or death, nine locally and nine distantly (including two patients with both local and distant recurrence), and four died. The four deaths were all with radiation‐induced second primary sarcomas: Three with disease progression at 5.7, 9.9, and 11.6 months after IMCT, respectively; another who experienced Grade 4 hemorrhage died of hemorrhage at 3.4 months after IMCT with unknown disease status. The 1‐ and 2‐year overall survival (OS), progression‐free survival (PFS), local recurrence‐free survival (LRFS), and DM‐free survival (DMFS) rates for the entire cohort were 92.9% vs 90%, 73.6% vs 57.4%, 88.4% vs 78.9%, and 84.6% vs 76.5%, respectively. Those rates for patients without prior RT were 100% vs 100%, 82.1% vs 65.8%, 93.6% vs 85.3%, and 88.4% vs 79.5%, respectively.

For the main pathology types, chondrosarcoma and rhabdomyosarcoma, accounting for nearly 60% of the entire cohort, the survival rates were calculated individually. The 1‐ and 2‐year OS, PFS, LRFS, and DMFS rates of chondrosarcoma were 94.1% vs 94.1%, 79.1% vs 79.1%, 89.7% vs 89.7%, 84.4% vs 84.4%, respectively; the 1‐ and 2‐year OS, PFS, LRFS, and DMFS rates of rhabdomyosarcoma were 100% vs 100%, 53.3% vs 17.8%, 87.5% vs 70.0%, 66.7% vs 44.4%, respectively.

### 3.5 Predictive factors for LRFS, DMFS, PFS, and OS

**Figure 2:**
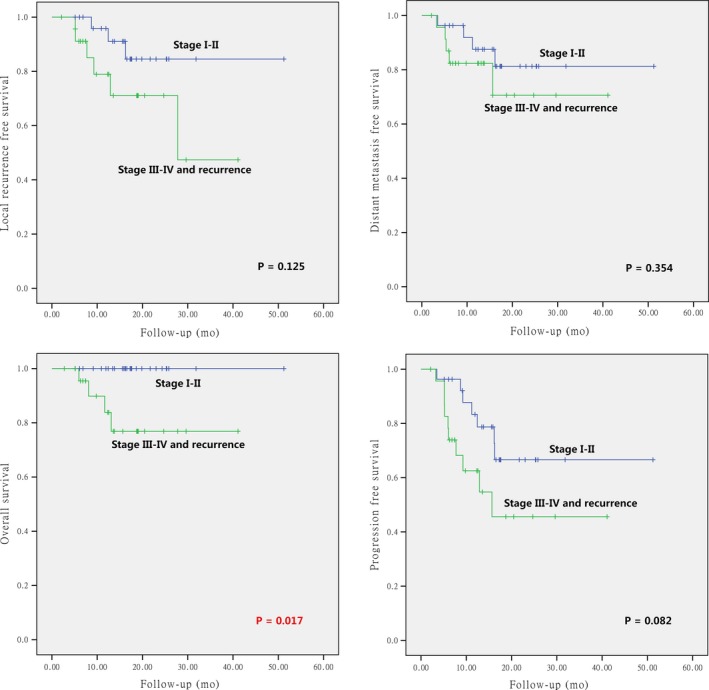
Local control, overall survival, distant metastasis‐free survival, and progression‐free survival according to clinical stages (clinical stage I ~ II vs clinical stage III ~ IV and recurrence) for the entire group of patients

**Figure 3:**
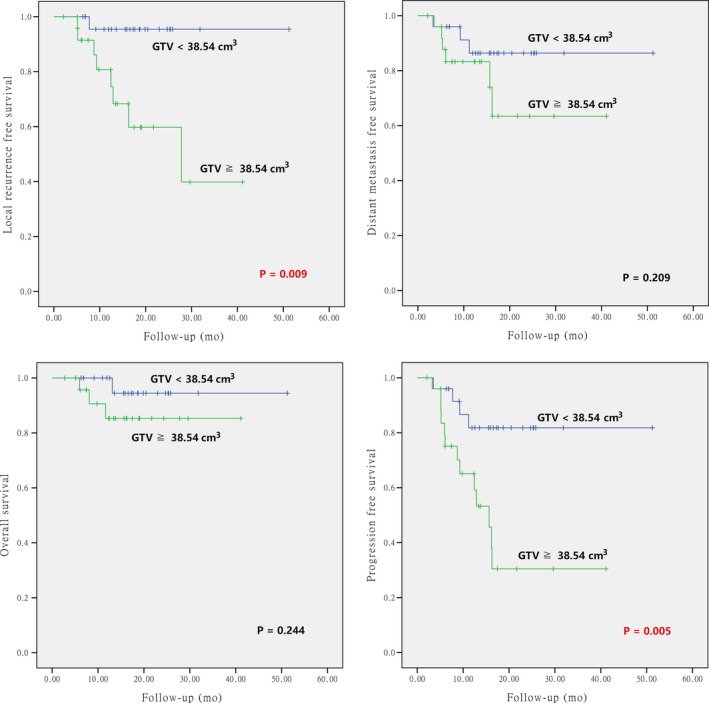
Local control, overall survival, distant metastasis‐free survival, and progression‐free survival according to the GTV volumes (< vs ≥ median volume)

**Figure 4:**
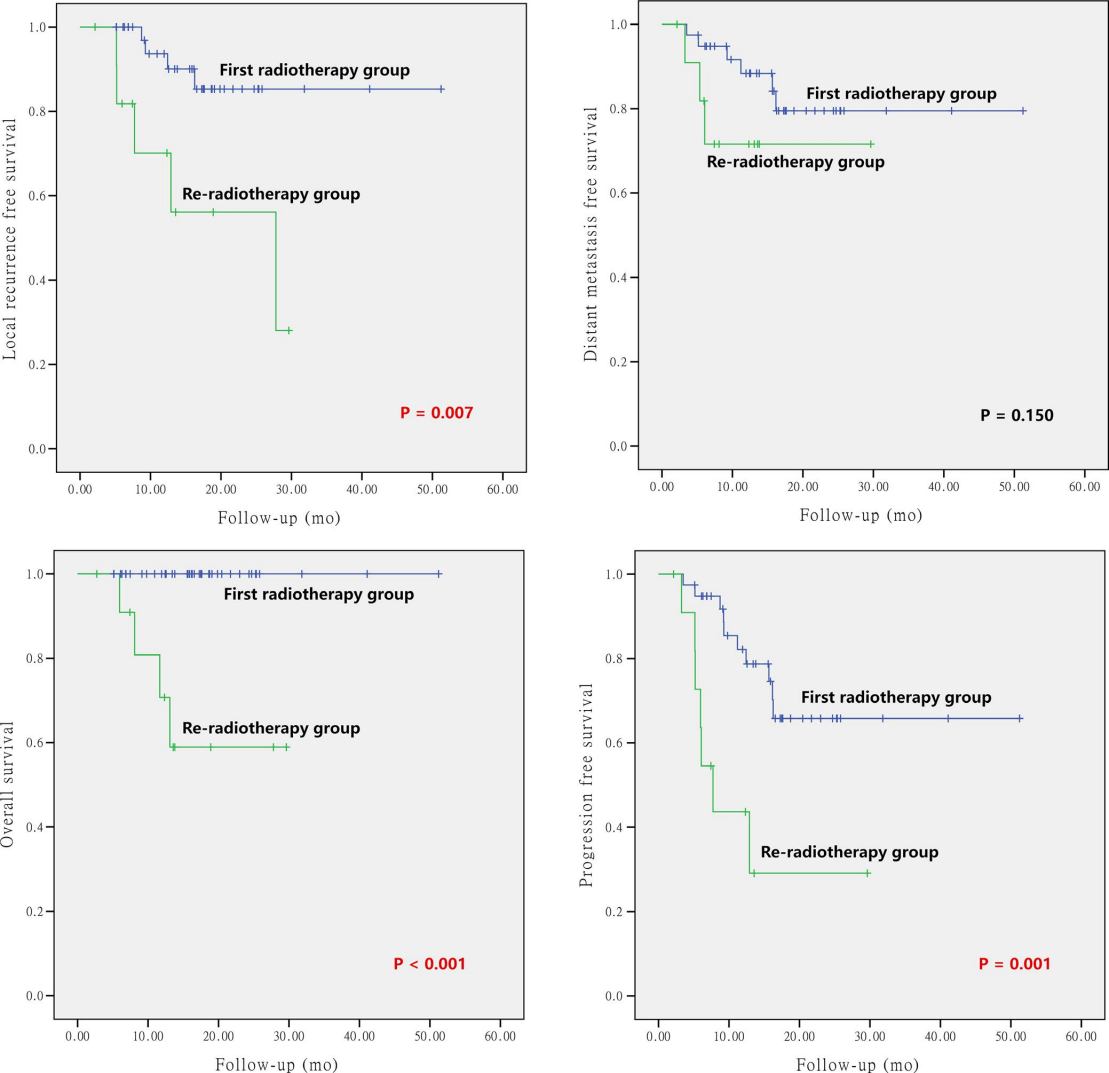
Local control, overall survival, distant metastasis‐free survival, and progression‐free survival according to initial vs re‐irradiation

**Figure 5:**
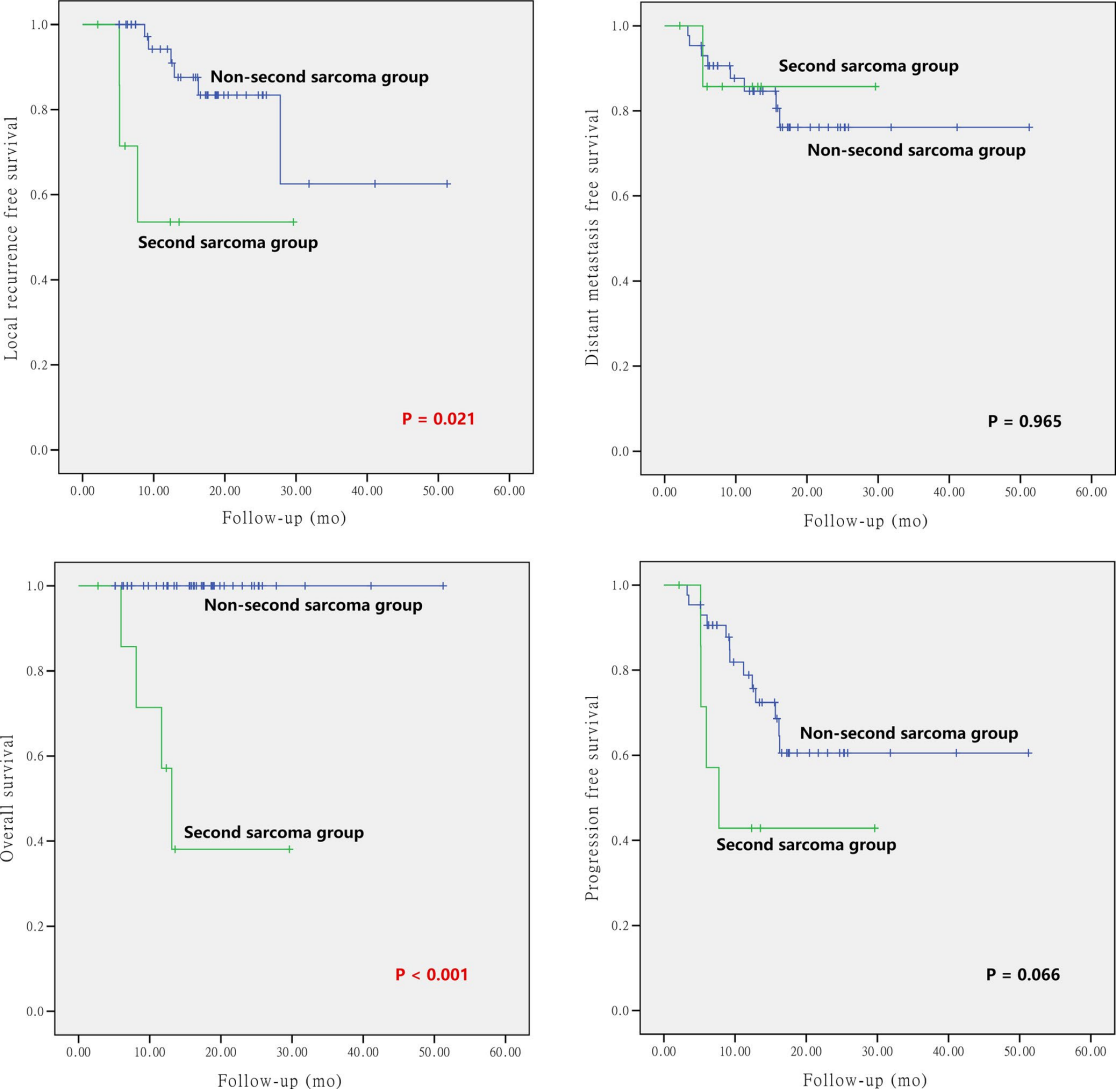
Local control, overall survival, distant metastasis‐free survival, and progression‐free survival according to primary vs radiation‐induced second primary head and neck sarcomas

The differences of the survival probabilities were compared by Log‐rank test in terms of sex, age, clinical stage, GTV volume, pathology, sites of disease origin, first time vs repeated radiotherapy, primary vs secondary sarcoma, and particle beam(s) used. The results revealed statistically significant differences in clinical stage for OS (*P* = 0.017), GTV volume for LRFS and PFS (*P* = 0.009 and 0.005, respectively), re‐radiotherapy for LRFS, PFS, and OS (*P* = 0.007, 0.001, and < 0.001, respectively), and second primary sarcoma for LRFS and OS (*P* = 0.021 and < 0.001, respectively). The results are detailed in Table 5 and Figures 2, 3, 4, 5.

**Table 5:** Univariate analysis for the 1‐year LRFS, DMFS, PFS, and OS of 51 cases of HNS

Factor	1y LRFS	*P* value	1y DMFS	*P* value	1y PFS	*P* value	1y OS	*P* value
Sex		0.634		0.870		0.563		0.861
Male	0.909		0.820		0.699		0.910	
Female	0.862		0.870		0.773		0.952	
Age		0.404		0.593		0.312		0.168
≤40	0.920		0.849		0.805		0.955	
>40	0.833		0.844		0.648		0.894	
Clinical stage		0.125		0.354		0.082		**0.017**
I ~ II	0.958		0.875		0.833		1.0	
III ~ IV + recurrent	0.790		0.824		0.625		0.839	
GTV volume*		**0.009**		0.209		**0.005**		0.244
<38.54 cm3	0.955		0.863		0.818		0.944	
≥38.54 cm3	0.807		0.833		0.651		0.853	
Pathology		0.140		0.498		0.064		0.416
Chondrosarcoma	0.897		0.844		0.791		0.941	
Non‐chondrosarcoma	0.874		0.849		0.698		0.920	
Site		0.435		0.247		0.057		0.906
Nasal cavity‐paranasal sinus	0.929		0.673		0.612		0.900	
Skull base	0.878		0.878		0.816		0.938	
Others	0.862		0.941		0.757		0.944	
Re‐radiotherapy		**0.007**		0.150		**0.001**		**<0.001**
Yes	0.701		0.716		0.436		0.701	
No	0.936		0.884		0.812		1.0	
Second primary		**0.021**		0.965		0.066		**<0.001**
Yes	0.536		0.857		0.429		0.571	
No	0.910		0.846		0.789		1.0	
PT beam types		0.715		0.528		0.497		0.510
IMCT	0.883		0.836		0.724		0.907	
IMPT + IMCT	0.875		0.875		0.750		1.0	
IMPT	1.0		1.0		1.0		1.0	

Abbreviations: DMFS, distant metastasis‐free survival; LRFS, local recurrence‐free survival; OS, overall survival; PFS, progression‐free survival; PT, particle therapy.

* The median volume of GTV was 38.54 cm^3^.

The predictive factors with a *P* value of 0.5 or less from the univariate analyses were included in the multivariate analyses using Cox regression for LRFS and PFS (Tables 6 and 7). As all patients with radiation‐induced second primary sarcomas received re‐irradiation, initial vs second primary sarcoma was excluded in the multivariate analyses to avoid duplicity. Multivariate analyses revealed that re‐irradiation was an independent prognostic factor for both LRFS and PFS (*P* = 0.015 and 0.037, respectively). In addition, GTV volume was significant for PFS (*P* = 0.048). No significant predictive factor was found for OS in multivariate analysis, potentially due to limited number of events. Multivariate analyses for DMFS was not performed as no significant outcome was observed in univariate analyses.

**Table 6:** Cox proportional hazard regression analysis for 1‐year LRFS

	Wald	Sig.	HR	95.0% CI for Exp(B)	
				Lower	Upper
Age	0.701	0.402	3.627	0.787	16.705
Clinical stage	0.006	0.940	0.518	0.047	5.712
GTV volume	3.545	0.060	7.659	0.799	73.371
Pathology	0.880	0.348	0.360	0.027	4.755
Site	0.661	0.416	1.225	0.311	4.831
Re‐radiotherapy	5.870	**0.015**	5.211	1.371	19.811

Abbreviations: CI, confidence interval; HR, hazard ratio; LRFS, local recurrence‐free survival.

**Table 7:** Cox proportional hazard regression analysis for 1‐year PFS

	Wald	Sig.	HR	95.0% CI for Exp(B)	
				Lower	Upper
Age	0.609	0.435	2.125	0.646	6.989
Clinical stage	0.000	0.986	0.628	0.146	2.704
GTV volume	3.893	**0.048**	3.265	1.008	10.579
Pathology	1.904	0.168	0.588	0.107	3.239
Site	2.265	0.132	1.538	0.591	4.004
Re‐radiotherapy	4.356	**0.037**	2.991	1.069	8.367
PT beam types	0.052	0.820	1.385	0.315	6.090

Abbreviations: CI, confidence interval; HR, hazard ratio; PFS, progression‐free survival; PT, particle therapy.

## 4 discussion

This study presents 51 consecutive, nonselected patients who received IMCT and/or IMPT for HNS. With a median follow‐up time of 15.7 months, we have reported a relatively favorable outcome for this challenging condition although the majority of patients had unresectable disease. The 1‐ and 2‐year OS rates were 92.9% and 90.0%, respectively, and those for patients who had no prior RT were both at 100%. All four deceased patients had radiation‐induced secondary sarcoma. Among them, one experienced acute Grade 4 event (hemorrhage) and eventually deceased from bleeding. Overall, the treatment appears to be well tolerated.

RT is an important local modality for the management of HNS. However, disease control after photon‐based RT is limited for both osteo‐ and soft‐tissue sarcomas. Except for the small cell variant, osteosarcoma is biologically resistant to photon‐based RT, which is usually inadequate to achieve local control, especially for tumors with large volume.^26^ Results of unresected or recurrent skull base chondrosarcoma treated with conventional photon‐based RT are also suboptimal.^27^ Long‐term control after conventional RT for unresectable soft‐tissue sarcomas of the head and neck is uncommon.^28^, ^29^

Oftentimes, RT is provided as an adjuvant treatment as the tumor proximity and involvement of critical head and neck structures preclude R0 resection with a wide margin. However, this anatomical reality also limits the RT dose that can be delivered for a curative intent, due to the dose constraints of the OARs. The use of adjuvant RT for HNS was largely derived from the clinical experience in extremity sarcomas, which argues in favor of adjuvant RT for high‐grade and large volume disease, or with positive and/or close margins after resection.^3^ Nevertheless, despite its prevailing utilization, the benefit of adjuvant RT for many subtypes of HNS is controversial, and results from retrospective reports are mixed. In the Surveillance, Epidemiology, and End Results (SEER) study of patients treated between 1973 and 2010, 11 481 adult and 1244 pediatric HNS cases were analyzed. Patients who received adjuvant RT resulted in a substantial reduction in survival as compared to those who did not receive radiation.^30^ However, the majority of patients who received adjuvant RT were those with clinically apparent high‐risk factors such as bulky and incompletely resected tumors, and a propensity‐matched model lately demonstrated no significant difference in cause‐specific survival between patients with or without adjuvant RT. Results of several retrospective studies indicated that the addition of adjuvant RT significantly improved local control, at least in patients with positive surgical margins or residual diseases.^28^, ^29^ As positive surgical margins were significant prognosticators in most studies,^29^, ^31^, ^32^ it is reasonable to recommend adjuvant RT to all patients unless R0 resection is confirmed. Nevertheless, although local failure has been considered as a significant negative indicator for survival, no study has confirmed that adjuvant RT significantly improved OS.

The unique physical characteristics of particle RT can obviate some of the limitations of photon‐based RT. The Bragg Peak phenomenon of PRT or CIRT allows little entry dose and a precise dose deposition to occur as the beam traverses through patient tissues. Following the Bragg Peak region along the beam path, there is a dose reduction step. Particle therapy is the superior technology used to treat the base of skull chordoma, chondrosarcoma, and other deeply located head and neck tumors, especially those within the vicinity of vulnerable tissues.^27^, ^33^ Furthermore, CIRT represents a high‐LET radiation, and the value of relative biological effectiveness (RBE) of CIRT is 3 ~ 5 (which is greater than proton or photon therapy), depending on the tumor or tissue type as well as the end point of study. As such, CIRT is theorized to be more effective in disease control of more radioresistant tumors, including many types of sarcomas, especially those patients who failed photon‐based RT.

There is a growing body of literature regarding sarcomas treated with CIRT. According to a retrospective study from the NIRS, 24 patients with retroperitoneal sarcoma were treated to 52.8 to 73.6 GyE of CIRT (passive scattering technology) in 16 fixed fractions over 4 weeks.^34^ The 2‐year OS and LC rates were 75% and 77%, respectively. No Grade 4 or Grade 5 GI complications were observed. In a phase I/II trial, Sugahara *et al* reported the results of CIRT in the treatment of 17 patients with soft‐tissue sarcoma of the extremities, including eight with recurrent disease after surgery with or without chemotherapy.^35^ None of these patients had prior RT. Mixed dose/fraction dose escalation schemes were used in the trial. The highest dose/fraction was 70.4 GyE (4.4 GyE/Fraction). The authors reported 3‐year OS and LC rates of 68% and 76%, respectively. Furthermore, no Grade 4 acute or late toxicity was observed. Similar results were reported for 47 patients with primary spinal sarcoma after CIRT.^36^ The 5‐year LC and OS rates were 79% and 52%, respectively, after CIRT to a median dose of 60.4 GyE in 16 fractions. Additionally, the use of CIRT was reportedly safe and effective for primary skull base chondrosarcomas. In 79 patients with base of skull chondrosarcomas, treated with CIRT to 60GyE (3 GyE/fraction), the 3‐year LC and OS rates of 95.9% and 96.1% were reported. Long‐term LC and OS were equally favorable. In addition, no radiation‐induced secondary malignancies were observed.

Particle RT has been used for the management of osteosarcoma as well. The 5‐year local control of 72% has been reported after proton therapy to a median of 68.4 GyE in a retrospective series.^37^ In a group of 78 patients with inoperable osteosarcoma of the trunk treated at NIRS using CIRT, a long‐term control rate of 62% was reported.^38^

It is difficult to compare our results with those from the above‐mentioned studies of particle RT due to limited number of patients with HNS included in those studies, the different techniques (IMCT with PBS technology versus passive scattering) used, and the relatively short time of follow‐up of our patients. Our multivariate analyses revealed that re‐irradiation was an independent prognostic factor for both LRFS and PFS. And, volume of the tumor (measured as GTV volume) was an independent prognostic factor for PFS. These observations are in consistent with most reports of HNS treated with photon‐based RT, although our LRFS and survival appeared substantially better. In an analysis of 65 patients with HNS, Le *et al* discovered that tumor size and grade were important predictors for local control.^28^ In another series of 46 patients with HNS (angiosarcomas excluded), T‐classification was a significant prognosticator for local control.^39^ No comparison in OS or its predictive factor could be made with any other studies since re‐irradiation was the only significant factor in our series, and all patients died at the time of this analysis had radiation‐induced secondary sarcoma. Nine patients experienced DM in our series, which is substantially higher than those reported in most published literatures. Unfortunately, we were not able to identify a significant predictive indicator for DMFS.

As far as we know there have been no published literature on the use of carbon‐ion or proton radiation for HNS except for limited reports on skull base chondrosarcoma. Our series included a relatively large number of patients for this rare disease category from a prospectively established registry and database. However, the study suffered from a relatively short follow‐up time. In addition, sarcoma is a disease group with close to 50 pathological entities. As such, our study is also hampered by a heterogeneous group of histological subtypes which included soft‐tissue sarcoma, chondrosarcoma, and osteosarcoma. The inclusion of recurrent disease after surgery or radiotherapy also complicated different biology and radiosensitivities. Given the rarity of HNS, most series on the topic were retrospective in nature and suffered from limited number of cases accrued over a long period of time. And, most of the results were published before the new millennium. It is unlikely that any prospective randomized clinical trials of this diagnosis could be conducted in the future. The development of new treatment technology or technique will largely depend on well designed and conducted phase I/II trials or retrospective studies.

## 5 CONCLUSION

With few observed acute and late toxicities, intensity‐modulated carbon‐ion or proton radiotherapy provided effective short‐term tumor control in the management of HNS. Further investigations, preferably prospective trials, will be required to confirm the efficacy and toxicities of IMCT in this group of patients. Given the relative rapidity of case accumulation within this study, that is, 54 patients over 3 years, a randomized phase II trial to investigate the effectiveness of additional high‐dose boost with carbon‐ion beam to the hypoxic region defined by functional imaging was planned.

## CONFLICT OF INTEREST

The authors have declared that no competing interest exists.
